# Prediction of COD in industrial wastewater treatment plant using an artificial neural network

**DOI:** 10.1038/s41598-024-64634-z

**Published:** 2024-06-14

**Authors:** Özgül Çimen Mesutoğlu, Oğuzhan Gök

**Affiliations:** Environmental Engineering Department, Aksaray University, Aksaray, Turkey

**Keywords:** Artificial neural network, Chemical oxygen demand, Wastewater treatment plant, Principal component analysis, Environmental sciences, Environmental chemistry

## Abstract

In this investigation, the modeling of the Aksaray industrial wastewater treatment plant was performed using artificial neural networks with various architectures in the MATLAB software. The dataset utilized in this study was collected from the Aksaray wastewater treatment plant over a 9-month period through daily records. The treatment efficiency of the plants was assessed based on the output values of chemical oxygen demand (COD) output. Principal component analysis (PCA) was applied to furnish input for the Feedforward Backpropagation Artificial Neural Networks (FFBANN). The model’s performance was evaluated using the Mean Squared Error (MSE), the Mean Absolute Error (MAE) and correlation coefficient (R^2^) parameters. The optimal architecture for the neural network model was determined through several trial and error iterations. According to the modeling results, the ANN exhibited a high predictive capability for plant performance, with an R^2^ reaching up to 0.9997 when comparing the observed and predicted output variables.

## Introduction

Water quality is a critical global concern, especially when considering the scarcity of clean water, necessitating the optimal utilization of limited resources. Pollution arising from the discharge of chemical pollutants in both industrial and domestic wastewaters poses a significant threat to aquatic biota^1^. Improper operation of wastewater treatment plants (WWTPs) can lead to environmental and public health problems. The discharge of treated effluent into water sources has the potential to spread disease and have adverse effects on aquatic ecosystems. Therefore, the implementation of effective monitoring and control techniques for wastewater systems is well-recognized by water and environmental engineers. However, modeling a WWTP presents challenges due to the complexity of the processes involved^2^. The relevant physical, biological, and chemical processes exhibit nonlinear behaviours, making them challenging to describe using conventional linear mathematical models^3^. Therefore, reliable and easy monitoring of treatment systems can be achieved by developing robust, nonlinear methods capable of predicting WWTP performance using measured values of wastewater parameters.

In recent years, the use of machine learning algorithms has become increasingly appealing as an alternative to the induction strategy for deriving models from experimental data in wastewater processes^4^. AI-based models are used to benchmark the performance of WWTPs, because their process control systems can be seamlessly integrated with AI models, enabling fully automated operation with minimal human intervention^5^. AI-based models for wastewater treatment systems can effectively handle the nonlinearity and complexity of these processes. In particular, the ANN model overcomes the limitations of earlier numerical and mathematical models developed for wastewater treatment systems^6^. There are many statistical and AI-based predictive models used for this purpose. Among the widely and successfully used methods are various Artificial neural networks (ANNs)^5^, response surface methodology (RSM)^7,8^, radial basis function (RBF) ^8^, and fuzzy logic^9^.

ANNs, as computational techniques, are nonlinear models designed to mimic the functionality and decision-making processes of the human brain^10^. ANNs have been increasingly applied in various environmental modeling studies^11,12^ and investigations into water quality issues^13,14^. In the domain of wastewater treatment plant (WWTP) modeling, ANNs have been successfully employed for the prediction of WWTP parameters^15–17^, process control^18–21^, and the estimation of output parameters and characteristics^22,23^. However, many of these studies necessitate diverse input data, contributing to the costliness and time-consuming nature of the modeling process.

This study demonstrated the use of an ANN for predicting and forecasting chemical oxygen demand (COD) within Aksaray Industrial wastewater Treatment Plant (AIWWTP), where intricate and dynamic processes are concealed within the monitored datasets. The training process focused on estimating and predicting parameters, specifically COD, by minimizing the error function while adhering to the permissible limits set by the AIWWTP license. This technique serves as a valuable complement to conventional mathematical modeling methods commonly employed for the prediction and forecasting of wastewater treatment plants.

## Materials and methods

### Principal component analysis (PCA)

PCA stands out as a widely adopted statistical technique in the domain of dimensionality reduction and multivariate data analysis and has gained increasing popularity over the past two decades across various fields^24^. Successful applications of PCA analysis have been observed in modeling diverse industrial processes, including the modeling of the IWWTP^25^ within this period. The fundamental principle behind PCA lies in leveraging the collinear nature of the data to effectively reduce the dimensionality of the measurement space. This is achieved by introducing a few essential pseudovariables known as principal components (PCs). These components serve to elucidate the primary mechanisms steering the underlying process and are typically fewer in number than the measured variables. PCA represents one of the multivariate statistical method that can simplify the complexity of input variables, particularly when dealing with extensive information volumes, aiming for an enhanced interpretation of variables^26^.

### Artificial neural network (ANN) model

ANNs, which are extensively employed for predicting valuable data from nonlinear variables, are shaped by three fundamental components: architecture, activation functions, and the training algorithms^27^. This network comprises input, hidden, and output layers. The input layer accepts variables and facilitates transmission, while the hidden layer conveys variations to the output layer. The output layer generates the final output of the structure. Each layer is interconnected by nodes (neurons), each performing a distinct nonlinear activation function. A hidden node produces an intermediate output by performing a weighted sum of inputs and then transforming it with a transfer function. Hidden nodes transfer data to connected nodes in the next layer until the output layer completes the process by producing the final output^28^. Factors such as the number of layers, neurons, and the type of activation function employed significantly influence the performance of the ANN model structure. However, careful consideration and selection of these parameters are crucial when constructing a model for specific applications^29–31^.

**Figure 1:**
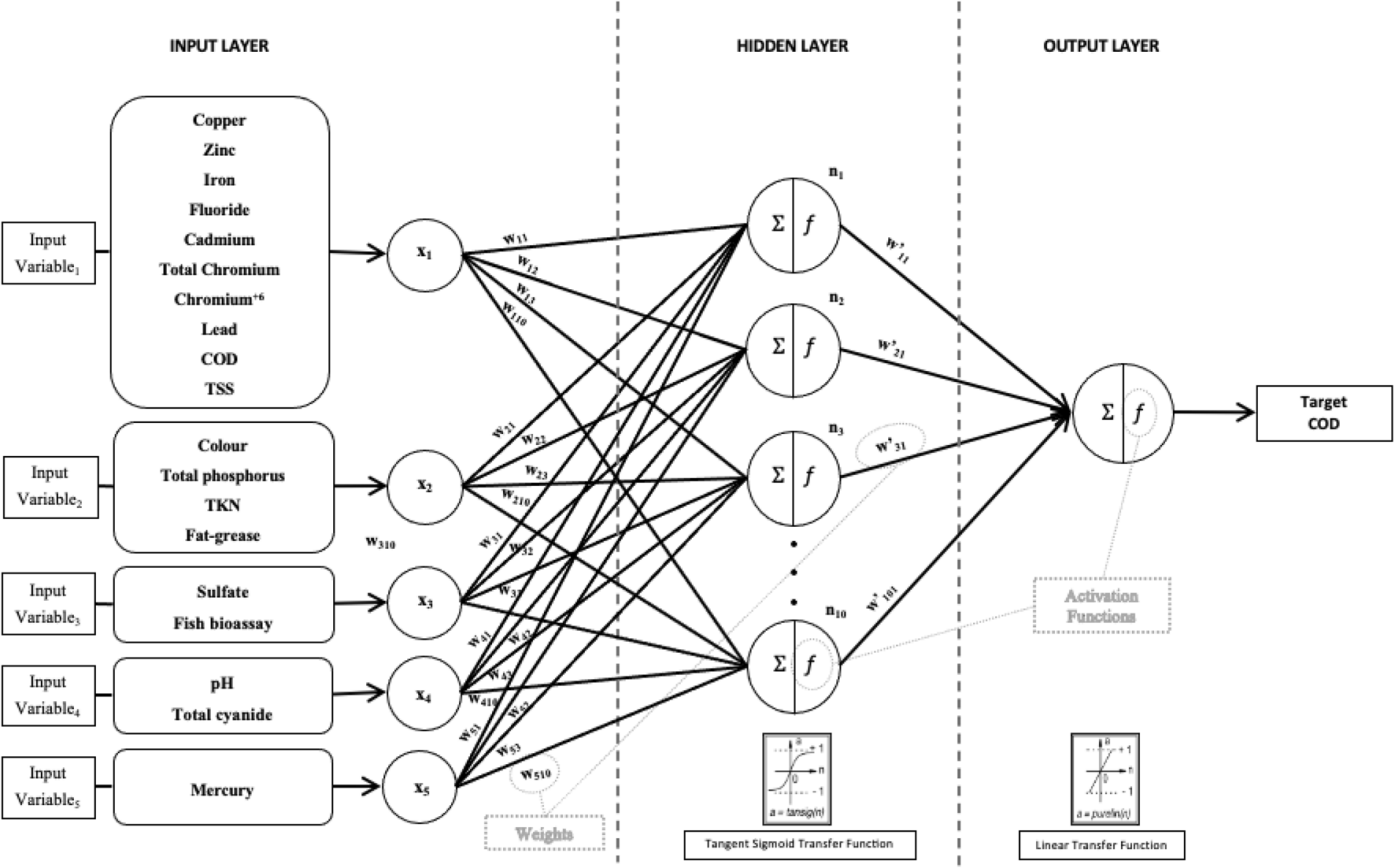
Schematic view of a feedforward neural network with an input layer, a hidden layer and an output layer.

ANNs represent a complex computational framework with a distributed nature, characterized by multiple processing elements operating concurrently. Within this intricately structured system, interconnected components possess the inherent capability to autonomously adapt their connection strengths during the learning process. The primary aim of this research was to predict COD concentrations in industrial wastewater by creating ANN structure using MATLAB (MATRIX LABORATORY) Online (basic version) mathematical software^32^. The ANN employed in this analysis consists of an input layer, a hidden layer, and an output layer, each comprising multiple neurons, as depicted in Fig. 1. To prevent numerical overflows from arising from excessively large or small weights, normalization of the input and output data was conducted, constraining them to a range between 0 and 1, as exemplified in Eq. (1)^33^:

1 $${\text{x}}_{norm}=\frac{{\text{x}}_{\text{i}}-{\text{x}}_{\text{min}}}{{\text{x}}_{\text{max}}-{\text{x}}_{\text{min}}}$$

In this context, the normalized value (x_norm_) is calculated based on the original data (x_i_) using the maximum (x_max_) and minimum (x_min_) values. This process ensures that the scaled data fall within the range of 0–1.

For this study, the FFBPANN algorithm, initially proposed by Rumelhart, Hinton, and Williams^27^ was employed. The FFBPANN algorithm is highly effective at learning in ANNs; it operates by propagating the error from the output layer back through the hidden layer and to the input layer of the network to achieve the desired final outputs. The algorithm utilizes the gradient descent technique to calculate the network's weight and adjust the interconnection weights to minimize the output error, as shown in Eq. (2)^34^:

2 $${W}_{ix}^{m}={W}_{ix}^{m-1}+{W}_{ix}^{m}={W}_{ix}^{m-1}+\eta \times {\delta }_{x}^{n}\times {A}_{i}^{n-1}$$

In Eq. (2), the connective weight (*W*_*ix*_) represents the weight associated with a particular connection, while η denotes the learning rate that influences the weight adjustment process. The error signal (${\delta }_{x}^{n}$) and the output value of the sublayer (${A}_{i}^{n-1}$) also play crucial roles in determining the new weight values. The summation function is employed to compute the weighted sum of all the input signals, serving as the initial step in the network's computation process, as described in Eq. (3)^35^:

3 $$f\left(x\right)={\sum }_{i=1}^{n}\left({W}_{ix}+{a}_{i}\right)$$

In this paper, a hyperbolic tangent sigmoid transfer function was used in the hidden layer and a linear transfer function was used for the output layer^36^. To determine the ideal architecture, neural networks were trained using varying iteration numbers (epochs). The dataset was subjected to random partitioning, resulting in three separate subsets: 70% for training, 15% for validation, and the remaining 15% for testing purposes.

### Study area and data collection

In this study, measurement data for wastewater parameters monitored at the AIWWTP were collected over a 9-month period. Wastewater samples for these parameters were collected daily by experts from the facility and analyzed in an accredited laboratory. A total of 19 parameters were utilized for the ANN. These parameters are listed in Table 1.

**Table 1:** Results of the AIWWTP measurement parameters.

Parameters	Unit	Max	Min	Mean
COD_Influent_	mg/L	2126	1357	1808
pH	–	7.98	7.01	7.41
Colour	mg/L Pt–Co	228.38	148.75	181.5
TSS	mg/L	1059.41	481.05	755.15
Copper	mg/L	0.08	0.02	0.06
Mercury	mg/L	0.0080	0.0001	0.0012
Zinc	mg/L	0.180	0.049	0.088
Iron	mg/L	4.874	1.727	3.019
Fluoride	mg/L	2.191	0.633	1.240
Cadmium	mg/L	0.006	0.001	0.003
Total Chromium	mg/L	0.974	0.097	0.317
Chromium^+6^	mg/L	0.097	0.018	0.057
Lead	mg/L	0.102	0.048	0.070
Sulfate	mg/L	1007	458	751
Total phosphorus	mg/L	9.23	3.97	6.52
TKN	mg/L	27.64	13.97	19.80
Total cyanide	mg/L	0.006	0.004	0.005
Fat-grease	mg/L	78	47	59
Fish bioassay	–	9	5	7
COD_effluent_	mg/L	193	10	71

### Model performance evaluation

The purpose of the performance evaluation of the trained ANN model was to assess the quality of the developed model. To achieve this, several statistical measurements were considered when evaluating the performance of the ANN model. These include the mean squared error (MSE) Eq. (4), the mean absolute error (MAE) Eq. (5) and the coefficient of multiple determination (R^2^) Eq. (6)^37^. These metrics offer insights into the model's predictive efficiency across different datasets; lower MSE and MAE values indicate better performance, while higher R^2^ scores suggest improved explanatory power.

4 $$MSE=\frac{1}{N}\sum_{t=1}^{N}{({Y}_{t}-{\widehat{Y}}_{t})}^{2}$$

5 $$MAE=\frac{1}{n}{\sum }_{i=1}^{n}\left|{y}_{i}-{\widetilde{y}}_{i}\right|$$

6 $${R}^{2}=1-\frac{\sum_{t=1}^{N}{({Y}_{t}-{\widehat{Y}}_{t})}^{2}}{\sum_{t=1}^{N}{({\widehat{Y}}_{t})}^{2}}$$

## Results and discussion

### Wastewater characteristics

Prior to predicting COD concentrations, the wastewater characteristics in the AIWWTP were consistently monitored daily from March to November 2023. The values of the monitored parameters are described in Table 1.

A wastewater treatment facility monitors various physical, chemical, and microbiological parameters^38^. Table 1 presents the parameters that are tracked daily, influencing COD_effluent_ and indicating treatment efficiency. Additionally, the facility conducts less frequent microbiological analyses. However, these analyses are performed primarily in instances of unusual declines in treatment efficiency and are therefore not considered within the model limits.

### PCA results

The way we choose the input parameters truly matters for how well the ANN model works. In this study, we used PCA to identify a smaller set of important new variables (components and dimensions) by combining different physical and chemical factors that might affect the amount of COD_effluent_. The PCA analysis is commonly employed with the aim of reducing the number of input variables provided to the ANN model and minimizing deviations in the dataset^39^.

**Figure 2:**
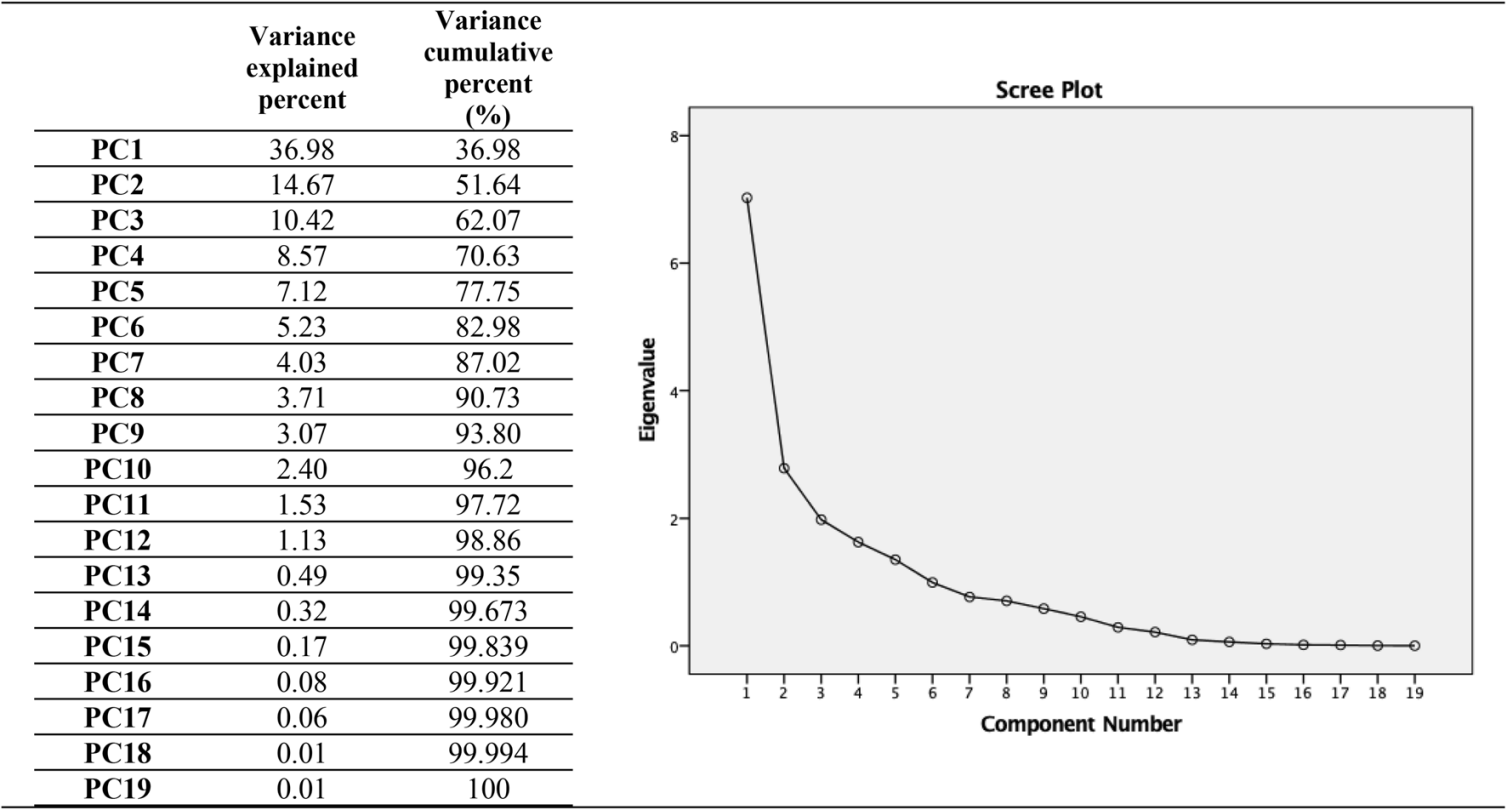
Total variance explained by PCA analyses.

PCA was used to calculate component scores for each analyzed parameter in order to predict COD concentrations. The PCA analysis, as illustrated in Fig. 2, revealed the components. Beyond the fifth point, the contribution of components to the variance decreases, and the contributions of additional variances become comparable to each other. Therefore, five variables were utilized as inputs for the ANN to predict COD concentrations. The first five components illuminate the variation in COD concentration examined in our study. In the subsequent step, component scores were determined for each water sample, and the score coefficient matrix is detailed in Table 2.

**Table 2:** Varimax component matrix.

	Component				
	PC1	PC2	PC3	PC4	PC5
Copper	0.604	− 0.331	0.225	0.267	0.210
Zinc	0.685	− 0.053	0.421	− 0.224	− 0.245
Iron	0.795	0.001	0.341	− 0.252	0.282
Fluoride	0.726	0.399	0.184	− 0.132	0.159
Cadmium	0.774	0.193	0.084	0.088	0.077
Total Chromium	0.832	− 0.097	− 0.341	− 0.298	0.085
Chromium^+6^	0.776	0.185	0.041	0.221	− 0.038
Lead	0.658	0.503	0.037	0.068	0.035
COD_influent_	0.651	0.170	0.548	0.060	− 0.054
TSS	0.529	0.135	0.421	− 0.494	− 0.032
Colour	0.084	0.859	0.094	0.332	0.178
Total phosphorus	0.568	0.603	0.204	− 0.134	0.221
TKN	0.328	0.795	0.234	− 0.159	− 0.155
Fat-grease	− 0.151	0.639	− 0.257	− 0.096	0.441
Sulfate	0.088	0.176	0.457	− 0.055	0.839
Fish bioassay	0.126	0.086	0.938	0.000	0.096
pH	0.223	− 0.079	− 0.013	0.757	0.039
Total cyanide	− 0.245	0.132	0.026	0.775	0.113
Mercury	− 0.202	− 0.084	0.204	− 0.364	− 0.800

The components were identified by considering component loadings greater than 0.45^40^. In the predicted first principal component (PC1), the copper, zinc, iron, fluoride, cadmium, total, chromium, chromium^+6^, lead, COD_influent_ and TSS parameters were grouped. Therefore, the first principal component primarily explained the variation in the COD_effluent_, the parameter under consideration in our study, accounting for 36.98% of the total parameter variance. The second principal component (PC2) encompasses color, total phosphorus, TKN and fat-grease parameters and contributed to 14.67% of the total variance. Moreover, the third principal component (PC3) included sulfate and fish bioassay parameters, explaining 10.42% of the overall parameter variance. PC4 involves pH and total cyanide content, whereas PC5 included the mercury content. In conclusion, the first five components collectively elucidate 77.95% of the total variance. The positive or negative component loadings of the input parameters provide insights into the direction of the impact on the COD_effluent_ parameter.

### ANN running

**Figure 3:**
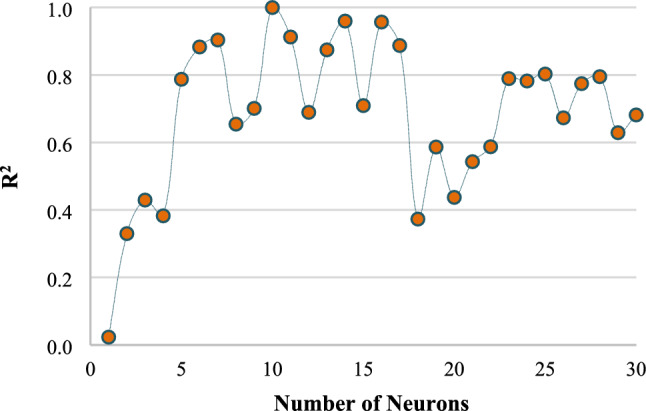
The R^2^ values across networks with varying numbers of neurons for the complete dataset.

**Figure 4:**
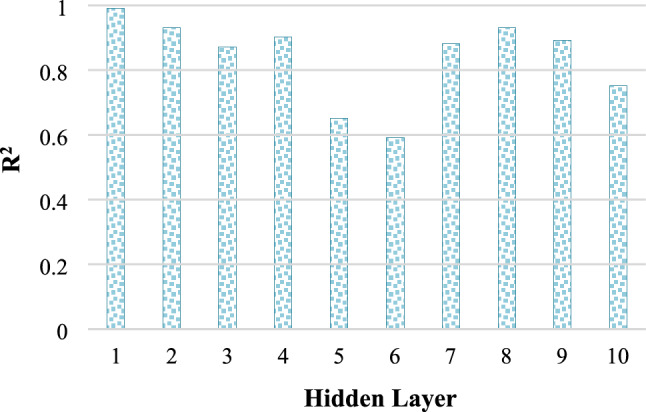
The R^2^ values across networks with different hidden layers.

In prediction models, the structure of the ANN model is crucial, and hence, the number of neurons in the hidden layer plays a significant role. Table 3 presents the results of the study conducted to determine the optimal number of neurons for the network structure, with R^2^, MSE and MAE outcomes obtained. In the study conducted as described in Fig. 3, utilizing a single hidden layer and employing the Levenberg–Marquardt (trainlm) training function, the R^2^ values corresponding to varying numbers of neurons are meticulously presented. Figure 4 illustrates how the predictive capacity of a FFBPANN with 10 neurons in each hidden layer is influenced by the number of hidden layers. The transfer function applied to neurons was the tangent sigmoid, while the training function utilized was trainlm. As demonstrated, the optimal prediction capability for the dataset was achieved with a single hidden layer.

**Table 3:** The results of the ANN for COD in the training stage with different architectures.

Name	Architecture*	Training function	Transfer function	Iteration	R^2^	MSE	MAE
M1	5-5-1	trainlm	tansig	28	0.7872	0.7632	0.6987
M2	5-6-1	trainlm	tansig	17	0.8829	0.0996	0.9109
M3	5-7-1	trainlm	tansig	54	0.9036	0.0988	0.1602
M4	5-8-1	trainlm	tansig	32	0.6542	0.2746	0.4328
M5	5-9-1	trainlm	tansig	12	0.7009	0.1405	0.2005
**M6**	**5-10-1**	**trainlm**	**tansig**	**16**	**0.9997**	**0.0625**	**0.0728**
M7	5-11-1	trainlm	tansig	64	0.9124	0.0933	0.2198
M8	5-12-1	trainlm	tansig	18	0.6892	0.8732	0.5692
M9	5-13-1	trainlm	tansig	19	0.8743	0.2091	0.4240
M10	5-14-1	trainlm	tansig	26	0.9596	0.0703	0.0325
M11	5-15-1	trainlm	tansig	33	0.7091	0.7543	0.5994
M12	5-16-1	trainlm	tansig	54	0.9568	0.0725	0.0581

*The first number is the number of input layer nodes; the second number is the number of hidden layer nodes and the last number is the number of output layer nodes.

Significant values are in bold.

**Figure 5:**
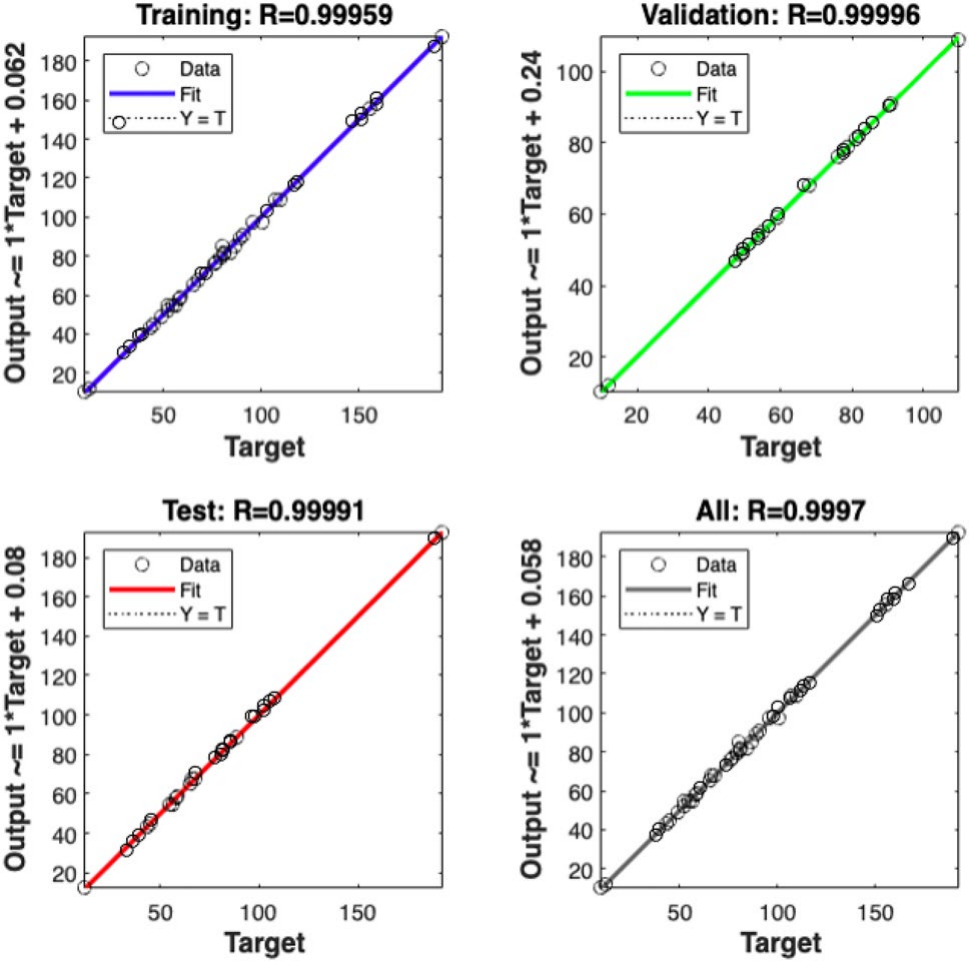
The correlation coefficients (R) for training, validation, testing, and overall results obtained from the analysis using the ANN structure.

In ANN studies, the process of achieving optimal learning and results close to the ground truth involves running the constructed network structure iteratively. During each iteration, the network generates an average mean squared error and correlation coefficient. The network's operation concludes when the model with the lowest mean squared error and the highest correlation coefficient is attained, and the network is then saved. In this research, Fig. 5 presents the regression results from the network that produced the lowest mean squared error and the highest correlation coefficient. The best determination coefficient was achieved when 70% of the data were used for training the neural network, 15% for validation, and 15% for testing. The R^2^ values obtained for training, validation, and testing were 0.99, 0.99, and 0.99, respectively.

**Figure 6:**
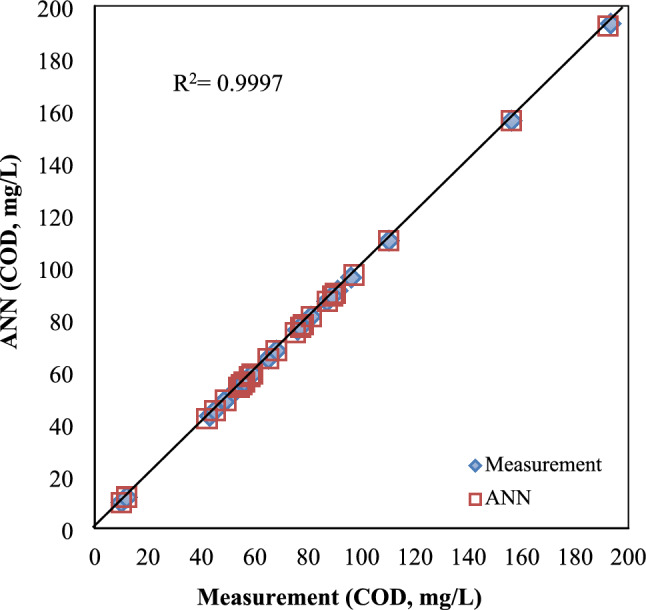
Correlation between the measured and predicted COD data.

As observed in Fig. 6, the comparison between the ANN predictions and the measured COD values demonstrates that the ANN prediction model performs exceptionally well. When comparing the values, the R^2^ value is calculated as 0.9997. This correlation coefficient indicates that the model is suitable for this study and can be reliably utilized in similar research. In the literature, Rashidi and Moghaddam^41^ used a multi-layer feedforward neural network for COD removal and developed a predictive model with a high correlation coefficient. Moghaddam et al.^42^ used a backpropagation neural network and found an R^2^ of 0.9843 for predicting the output parameter. In their study conducted at a wastewater treatment facility, Khatri et al. ^5^ compared various ANN models. The best predictive model was found to be the deep feedforward backpropagation (DFFBP) algorithm, which was implemented with 3 hidden layers and 11 neurons. This model achieved an initial R correlation coefficient of 0.997 on the training data set.

In both the world and our country, wastewater from various sectors is pre-treated before entering organized industrial wastewater treatment facilities, resulting in a homogenous pollution load based on the monitored parameters. Consequently, the flow schemes of industrial wastewater treatment plants are generally similar. In our study, the ANN model, which exhibits high predictive capability, can be applied to numerous domestic-industrial wastewater treatment facilities. For specialized industrial wastewater treatment plants, ANN prediction can be conducted using parameters specific to that industry.

## Conclusions

In the present investigation, the FFBPANN model was applied to determine the daily COD_effluent_ concentrations (R^2^) for the year 2023 at the inlet of the AIWWTP. Notably, the obtained R^2^ value for COD_effluent_ was 0.9997, with an associated MSE value of 0.0624, revealing the efficacy of the network structures. The optimal performance was achieved with the 5-10-1 ANN model, demonstrating its superiority in both the test and training datasets. These outcomes underscore the remarkable success of the ANN model employed in this research. Furthermore, the application of ANNs in the COD estimation was identified as a crucial aspect of PCA analysis aimed at determining the most effective model. The effectiveness of the FFBPANN model relies significantly on the input parameters, leading to superior results compared to traditional models.

Well-trained ANN parameters are crucial for providing reliable predictions in the wastewater treatment processes employed in WWTPs. In this study, it was concluded that the ANN model is successful at predicting the COD levels of WWTPs in terms of reliable and realistic results.

## Data Availability

The datasets used and/or analysed during the current study available from the corresponding author on reasonable request.
